# Nonsteroidal Anti-Inflammatory Drug-Induced Gastroduodenal Bleeding: Risk Factors and Prevention Strategies

**DOI:** 10.3390/ph3072225

**Published:** 2010-07-14

**Authors:** Marino Venerito, Thomas Wex, Peter Malfertheiner

**Affiliations:** Department of Gastroenterology, Hepatology and Infectious Diseases, Otto-von-Guericke University, Leipziger Str. 44, D-39120 Magdeburg, Germany; E-Mails: m.venerito@med.ovgu.de (M.V.); thomas.wex@med.ovgu.de (T.W.)

**Keywords:** NSAIDs, gastroduodenal bleeding, risk factors, prevention strategies

## Abstract

Nonsteroidal anti-inflammatory drugs (NSAIDs) are the most widely prescribed medications in the World. A frequent complication of NSAID use is gastroduodenal bleeding. Risk factors for gastroduodenal bleeding while on NSAID therapy are age, prior peptic ulcer and co-medication with anti-platelet agents, anticoagulants, glucocorticosteroids and selective serotonin-reuptake inhibitors (SSRI). Prevention strategies for at-risk patients include the use of the lowest effective dose of NSAIDs, co-therapy with proton-pump inhibitors and/or the use of a COX-2 selective agent. Treatment of *Helicobacter pylori* infection is beneficial for primary prophylaxis of NSAID-induced gastroduodenal bleeding in NSAID-naive patients. For patients with cardiovascular risk factors requiring NSAIDs, naproxen should be selected. In very high risk patients for both gastrointestinal and cardiovascular complications NSAID therapy should be avoided altogether.

## 1. Introduction

NSAIDs are the most widely used medications worldwide [1]. These drugs exert anti-inflammatory, antipyretic and analgesic effects by inhibiting cyclooxygenases (COX-1 and COX-2), the enzymes responsible for the production of prostaglandins, prostacyclins and thromboxanes. The beneficial actions of NSAIDs have been linked to their ability to inhibit inducible COX-2 activity, while side effects (e.g., gastrointestinal damage) are thought to be mediated by inhibition of COX-1 [2,3]. NSAIDs are classified in non-selective NSAIDs (inhibition of both COX-1 and COX-2) and COXibs (selective inhibition of the isoenzyme COX-2). Differently from all other NSAIDs, aspirin has the peculiarity of inhibiting COX enzymes irreversibly. Aspirin effects vary with dose [4]. Particularly at low doses (75–325 mg/day), aspirin preferentially acetylates serine 529 of COX-1 leading to an inhibition of platelet-derived thromboxane A2 [5]. This anti-thrombotic effect is routinely used in the prophylaxis of cardiovascular and cerebrovascular events, and results mostly from the fact that after the irreversible inhibition of COX-1, platelets are unable to newly synthesize COX-1 [6]. Therefore, the inhibition of platelets remains for their life-cycle. Table 1 shows a classification of NSAIDs according to the selectivity for COX enzymes and the type of COX inhibition (reversible/irreversible).

**Table 1:** Classification of NSAIDs according to the selectivity for COX enzymes and the type of COX inhibition (reversible/irreversible).

	Selective/non-selective inhibition of COX isoenzymes	Reversible/irreversible inhibition of COX isoenzymes
**NSAIDs**	non-selective inhibition of both COX isoenzymes	reversible
**COXibs**	Selective inhibition of COX-2	reversible
**ASA**	non-selective inhibition of both COX isoenzymes	irreversible

NSAIDs: traditional non-steroidal anti-inflammatory drugs, COXibs: NSAIDs that were specifically “designed” to selectively inhibit COX-2; ASA: aspirin.

NSAID intake increases the risk of developing peptic ulcers and their complications (bleeding, perforation or obstruction). Indeed, the risk of upper gastrointestinal bleeding in NSAID users has been shown to be approximately 4-fold higher than in people not taking NSAIDs [7] with an annual event rate per 1,000 persons of 13.6 (absolute risk frequency per year = 74) [8]. Gastric ulcers are approximately four times more common than duodenal ulcers in patients taking NSAIDs [9]. The risk of upper gastrointestinal complications increases also with the regular intake of aspirin at low-doses. Indeed, in a case-control study the risk of hospitalization for bleeding peptic ulcer with the current prophylactic aspirin regimens was 2.3 with the intake of 75 mg aspirin/day and increased to 3.2 and 3.9 with the intake of 150 mg/day and 300 mg/day, respectively [10]. In a meta-analysis of 14 placebo-controlled trials on vascular protection the absolute rate increase risk of major gastrointestinal bleeding with low-dose aspirin above placebo was 0.12% per year (95% CI: 0.07–0.19%) with a number-needed-to-harm of 833 patients (95% CI: 526–1429) [11].

## 2. Risk Factors for NSAID-Induced Gastroduodenal Bleeding

Risk factors for gastroduodenal bleeding in patients on NSAID therapy are age, prior peptic ulcer and co-medication with anti-platelet agents, anticoagulants, glucocorticosteroids and selective serotonin-reuptake inhibitors (SSRI) (Table 2). 

**Table 2:** Risk Factors for NSAID-associated gastroduodenal bleeding.

Risk factors	OR (95% CI)
Prior ulcer or ulcer complication [12]	4.76 (4.05–5.59)
Advanced age (60 years or older) [12]	5.52 (4.63–6.60)
Co-medication with	
- anti-platelet agents (ASA, Clopidogrel™l) [25]	7.4 (3.5–15)
- anticoagulants [19]	9.7 (4.6–20.2)
- glucocorticosteroids [12]	1.83 (1.20–2.78)
- SSRI [20]	12.2 (7.1–19.5)
*H. pylori* infection [57]	6.13 (9.98–373)

ASA: aspirin, SSRI: selective serotonin-reuptake inhibitors

The risk for NSAID associated gastroduodenal bleeding may further increase in the presence of more than one risk factor.

A history of peptic ulcer or ulcer complications have consistently emerged as important risk factors for NSAID-associated gastroduodenal bleeding. In a meta-analysis of 10 case-control or cohort studies the odds ratio (OR) for a first gastroduodenal bleeding with NSAID treatment was 2.39 (95% confidence interval CI, 2.16–2.65), whereas the risk in patients with a prior (or unspecified) history of a GI event increased to 4.76 (95% CI, 4.05–5.59) [12]. In a nested case-control study including 2,105 cases and 11,500 controls, patients on NSAIDs with a previous ulcer complication (bleeding or perforation) presented the greatest absolute risk of upper gastrointestinal bleeding with an incidence rate between 20 and 30 per 1000 person-years [13].

In the same study, the risk of developing upper gastrointestinal bleeding was found to depend also on dosage of NSAIDs (medium daily dose: RR = 2.4, 95% CI = 1.9–3.1; high daily dose: RR 4.9, 95% CI = 4.1–5.8) as well as on the pharmacokinetic of the NSAID used (plasma half-life less than 12 hours: RR = 3.1, 95% CI = 2.5–3.8; plasma half-life greater than 12 hours: RR = 4.5, 95% CI = 3.5–5.9) [13].

Advanced age is also a substantial risk factor. Although there does not appear to be a threshold age at which risk dramatically increases, the relative risk for developing a gastroduodenal bleeding increases linearly at the rate of approximately 4% per year of advanced age [14]. A systematic review of 18 case-control and cohort studies on serious gastrointestinal tract complications, showed an increased absolute risk of upper gastrointestinal bleeding among patients older than 75 years taking NSAIDs, with an absolute incidence rate around 20 per 1,000 person-years [15].

The risk of NSAID-induced gastroduodenal bleeding increases further with the intake of more than one NSAID or the combined medication with anti-platelet agents, anticoagulants, glucocorticoids and selective serotonin-reuptake inhibitors. Different studies have shown that a co-therapy with low-dose aspirin further increases the risk of developing a gastroduodenal bleeding of 2- to 6-fold in patients on NSAIDs [16,17,18,19]. Also the co-medication with anticoagulants has been shown to further increase the risk of gastroduodenal complications. In a case-control study conducted in Spain the OR for the development of a gastroduodenal bleeding was as high as 9.7 (95% CI 4.6–20.2) in patients receiving NSAIDs and vitamin K antagonists [20]. 

In a population-based study conducted in Denmark on 26,005 patients on SSRI (selective serotonin reuptake inhibitors) over four years, the risk of gastroduodenal bleeding was found to be 3.6-fold higher than expected and the concomitant use of SSRI and either NSAID or low-dose aspirin further increased the risk to 12.2 (95% CI, 7.1–19.5) and 5.2 (95% CI, 3.2–8.0), respectively [21]. The mechanisms by which SSRI aggravate NSAID-induced gastroduodenal injury have been poorly investigated. In an animal model the SSRI Paroxetine™ increased the severity of indomethacin-induced antral ulcers by impairing anti-oxidative systems (decrease in gastric mucosal superoxide dismutase and glutathione content) [22].

Mono-therapy with glucocorticosteroids does not increase the risk of a gastroduodenal bleeding [23]. However a meta-analysis of 16 studies demonstrated that the combined use of NSAIDs and glucocorticosteroids is associated with an increased risk of gastroduodenal bleeding (OR 1.83 95 % CI: 1.20 to 2.78) [12]. Studies on animal models suggest that glucocorticosteroids aggravate NSAID-induced gastroduodenal injury by inhibiting mucosal prostaglandin synthetase, thus further decreasing the biosynthesis of gastro-protective prostaglandins [24]. Furthermore 24-h pre-treatment with dexamethasone inhibited the peroxidase activity of COX enzymes by 83% in Wistar rats leading to an increase of reactive hydroxyl radicals that subsequently damage gastroduodenal mucosa [25]. 

Low-dose aspirin represents the standard therapy for the secondary prophylaxis of cardiovascular events. Current guidelines recommend the use of a double-antiplatelet therapy with aspirin and clopidogrel in patients with acute coronary syndrome or receiving a coronary artery stent. However in a population-based case-control study (1,443 cases of serious upper gastrointestinal bleeding during 2000–2004, 57,720 age and sex matched controls), patients receiving a combined therapy with low-dose aspirin and clopidogrel had an OR for developing a gastroduodenal bleeding as high as 7.4 (95% CI, 3.5–15) [26].

Thus, current data suggest that patients on NSAIDs with previous peptic ulcer or ulcer complications, age (>60 years) or combined therapy with anti-platelet agents, anticoagulants, glucocorticosteroids and SSRI should be considered as patients at increased risk for the development of a gastroduodenal bleeding. 

## 3. Strategies for Preventing Gastroduodenal Bleeding in Patients on NSAIDs

The first option for reducing GI toxicity of NSAIDs is to avoid medications that increase the risk of NSAID-induced gastroduodenal bleeding. Therefore, wherever possible the intake of drugs like anticoagulants, glucocorticosteroids and SSRI should be avoided. Moreover, a single NSAID should be prescribed using the lowest effective dose. The use of proton pump inhibitors (PPI) has been shown to significantly reduce endoscopic (RR 0.37, 95% CI 0.3–0.5) and symptomatic ulcers (RR 0.09, 95% CI 0.02–0.47) in patients on NSAIDs [27], and is therefore likely to be effective for primary prevention of NSAID-induced ulcer bleeding in patients with risk factors. There is no evidence for the superiority of one PPI over another or for high-dose of PPI over low-dose for preventing NSAID-induced gastroduodenal bleeding [28]. Therefore, a standard once-daily dosage of the cheapest PPI should be prescribed.

Switching to a COXib represents another option for reducing the risk of a gastroduodenal bleeding in patients on NSAIDs. Indeed the use of COXibs including the newest Lumiracoxib™ and Etoricoxib™ decreased the risk for serious gastrointestinal complications when compared with non-selective NSAIDs by 8 cases for every 1,000 patients treated with COXibs [8,17,27,29].

However, the 6-month rate of relapse for patients with previous gastroduodenal bleeding is still high, even if the patients take non-selective NSAIDs plus PPI or a COXib [30,31]. In a randomized controlled trial the combination of both strategies (COXib plus PPI) was evaluated [32]. Patients, taking NSAIDs for arthritis and admitted to the hospital due to acute gastroduodenal bleeding, were randomized to receive either Celecoxib™ 200 mg twice/day with Esomeprazole™ 20 mg twice/day or Celecoxib™ 200 mg twice/day with placebo. The combination treatment was found to be more effective than Celecoxib™ alone for prevention of ulcer bleeding in patients at high risk (13-month cumulative incidence of recurrent gastroduodenal bleeding was 0% in the combined-treatment group and 8.9% in the controls (95% CI: 4.1 to 13.7; p = 0.0004). Therefore, the combination of a COXib plus a PPI appears to be the safest option in patients at highest risk for developing a gastroduodenal bleeding. However, in very high-risk patients, if possible NSAIDs should be avoided altogether. 

## 4. NSAIDs Therapy: Balancing Gastrointestinal and Cardiovascular Risk

The use of COXibs or traditional NSAIDs, with the possible exception of naproxen, has been associated with an increased risk of serious cardiovascular events [33,34,35,36,37,38]. As a consequence, in April 2005, the US Food and Drug Administration (FDA) mandated that all NSAIDs should include a “black box” warning to highlight the potential increase in the risk of serious cardiovascular thrombotic events, along with the warning about potentially life-threatening gastrointestinal bleedings [available at http://www.fda.gov/cder/drug/ infopage/cox2/default.htm]. This means that before prescribing a NSAID, a multitude of gastrointestinal and cardiovascular factors needs to be considered. In a recent international workshop on gastrointestinal and cardiovascular effects of NSAIDs, clinical recommendations were generated by a panel of 19 experts for safer NSAID-treatment of patients with different levels of gastrointestinal and cardiovascular risks [39]. High gastrointestinal risk was defined as age ≥70 year, a positive history of a former upper gastrointestinal event (e.g. bleeding, ulcer) and/or the concomitant use of low-dose aspirin, glucocorticosteroids or anticoagulants. High cardiovascular risk was defined as established coronary artery disease, any cardiovascular disease that required prophylactic low-dose aspirin, or an estimated 10-year cardiovascular risk greater than 20%. The panel of experts recommended that patients with high cardiovascular risk requiring NSAIDs should be prescribed naproxen, whereas in high risk patients for both gastrointestinal and cardiovascular complications, NSAIDs should be avoided (Table 3).

**Table 3:** Algorithm for NSAID prescription based on gastrointestinal and cardiovascular risk factors.

	Low-risk for GI complications	High-risk for GI complications
**Low-risk for CV events**	non-selective NSAIDs	non-selective NSAIDs + PPI or COXibs + PPI
**High-risk for CV events/ ASA medication**	naproxen + PPI	avoid NSAIDs naproxen + PPI

GI: gastrointestinal, CV: cardiovascular, ASA: low-dose aspirin

## 5. Prevention of Gastroduodenal Bleeding in Patients on Double Antiplatelet Therapy

Current guidelines recommend a double antiplatelet therapy with low-dose aspirin plus Clopidogrel™ for ≥1 month after implantation of a bare metal coronary artery stent, ≥1 year after a drug-eluting stent, ≥1 month and 1 year after unstable angina or non-ST-elevation myocardial infarction managed without intervention, and 1 year after ST-elevation myocardial infarction [40,41,42]. In particular, the double antiplatelet therapy after implantation of a coronary artery stent has a crucial role in preventing a stent-thrombosis, an event associated with mortality rates of 20–45% [43]. The combined intake of low-dose aspirin and Clopidogrel™ is associated with an increased risk of gastroduodenal bleeding, with a number-needed-to-harm of 124 patients [26]. Therefore, the prescription of PPI in these patients has been recommended by the ACCF/ACG/AHA *Expert Consensus Document* [44]. Indeed, a gastroduodenal bleeding in these patients would have the clinical consequence of discontinuing at least the Clopidogrel™ therapy which in turn would increase the risk of a stent thrombosis. However, observational studies have shown that the use of PPI in patients taking Clopidogrel™ is associated with a small but statistically significant increased risk of re-admission or death for acute coronary syndrome (highest OR 1.40, CI 95% 1.10–1.77) [45,46,47]. As both Clopidogrel and PPIs are converted to their active metabolites by cytochrome P450 (CYP2C19) it has been hypothesized that the intake of PPIs might interfere with the activation of Clopidogrel™. As a consequence, the regulatory authorities FDA and EMEA have issued recommendations, discouraging concomitant use of PPI and Clopidogrel™-containing medicines (available online: http://www.fda.gov/Drugs/DrugSafety/PostmarketDrugSafetyInformationforPatientsandProviders/DrugSafetyInformationforHeathcareProfessionals/ucm190784.htm and http://www.emea.europa.eu/humandocs/PDFs/EPAR/Plavix/32895609en.pdf]. However, two prospective randomized controlled studies did not confirm a negative influence of PPI use on the clinical efficacy of Clopidogrel™ [48,49]. Thus, the current clinical evidence does not support the conclusion that PPIs increase the risk of cardiovascular events in all patients on Clopidogrel™. Moreover, *ex-vivo* pharmacodynamic studies using the platelet reactivity index (PRI) or the ADP-induced platelet aggregation as surrogate markers for the levels of platelet aggregation reported a reduced effect of Clopidogrel™ in patients on Omeprazole™, but not in patients taking other PPIs [50,51,52]. 

According to the current evidence and international expert recommendations, PPIs (*i.e.*, Omeprazole™) with the highest potential of interaction with Clopidogrel™ should be avoided in patients on Clopidogrel™ therapy. Furthermore, as both medications have very short half-lives, it may be appropriate to give one drug in the morning and the other in the evening to prevent possible interactions [53,54,55]. 

## 6. The Role of *H. pylori* Infection in NSAID-Induced Gastroduodenal Bleeding

*H. pylori* and NSAIDs are the most important risk factors in the pathogenesis of peptic ulcers and ulcer bleeding [56]. A meta-analysis of observational studies showed that the risk of developing gastroduodenal ulcer bleeding in *H. pylori-*infected patients on NSAIDs was 6.13, which was almost the sum of the two individual ORs estimated for *H. pylori* infection and NSAID use alone [57]. In a meta-analysis on the efficacy of *H. pylori* eradication in NSAID users has been shown that eradication therapy is beneficial for primary prevention of gastroduodenal ulcers (OR 0.26, 95% CI: 0.14–0.49), but does not reduce the risk of a gastroduodenal ulcer/ulcer bleeding relapse (OR 0.95, 95% CI: 0.53–1.72) in patients on NSAIDs [58]. A possible explanation for this phenomenon comes from the HELP NSAIDs study, in which the type of lesions having affected the patients over the previous 5 years was the most important determinant of the site of ulcer recurrence, irrespective of whether *H. pylori* was eradicated or not. This suggests that once a substantial mucosal injury has occurred, local mucosal factors are determinants for relapse, and the *H. pylori* status becomes less relevant [59].

However this may not be true when low-dose aspirin intake is considered. In a study by Chan *et al.*,250 *H. pylori*-positive subjects on low-dose aspirin with upper gastrointestinal bleeding were randomly assigned to administration of 20 mg Omeprazole™ daily for six months or one week of eradication therapy [60]. No significant differences were found between eradication therapy for *H. pylori* infection and maintenance treatment with Omeprazole™ for preventing recurrent bleeding in patients on low-dose aspirin. Indeed, only three patients had recurrent bleeding: two had been assigned to eradication therapy, and one to Omeprazole™. Noteworthy, one of the two patients who had recurrent bleeding after the eradication of *H. pylori*, took a concomitant NSAID for musculoskeletal pain. Thus, although the study was grossly underpowered for any definitive conclusion, both strategies appear to be effective for secondary prevention of low-dose aspirin induced ulcer bleeding, provided that other risk factors like concomitant NSAID intake are excluded. One likely explanation for the different effect of *H. pylori* eradication on ulcer relapse in NSAID users and low-dose aspirin users is that low-dose aspirin may not be as ulcerogenic as NSAIDs and induces bleeding mostly in pre-existing *H. pylori*-related ulcers. Thus, healing peptic ulcers by *H. pylori* eradication might be a potential option to reduce the risk of bleeding relapse in patients on long-term low-dose aspirin intake. However, currently available data are insufficient to answer this question. Therefore, a trial with a similar study design and a placebo arm is needed for definitive conclusions on this issue. No studies evaluating *H. pylori* eradication for primary prevention of low-dose aspirin-induced gastroduodenal ulcer bleeding are available. Taking in consideration the high mortality carried by a coronary artery stent thrombosis and the beneficial effect of *H. pylori* eradication on recurrent ulcer bleeding in patients on low-dose aspirin we suggest to test and treat for *H. pylori* infection all patients receiving combined therapy with low-dose aspirin and Clopidogrel™ after implantation of a coronary artery stent. Whether this option may represent a good alternative to PPI co-medication for preventing gastroduodenal bleeding in patients on double antiplatelet therapy still needs to be analyzed in a prospective randomized controlled trial. 

## 7. Conclusions

A multitude of gastrointestinal and cardiovascular risk factors should be considered before prescribing NSAIDs including low-dose aspirin. *H. pylori* test and treat strategy should be performed before starting a long-term NSAID-therapy. Whether this strategy is cost-effective, depends on the prevalence of *H. pylori* infection in the different countries, the efficacy of treatment available regimens and demographics. Prevention strategies for patients at high risk for gastrointestinal complications include the use of the lowest effective dose of NSAIDs, co-therapy with PPIs and/or the singular use of a COXib. For patients with cardiovascular risk factors requiring NSAIDs, naproxen should be selected. In patients on combined therapy with low-dose aspirin and Clopidogrel™ at increased risk for gastroduodenal bleeding, PPIs such as Omeprazole™ with the highest potential of interaction with Clopidogrel™ should be avoided and medications should be given with a separation of 12–15 hours (*i.e.*, PPI before breakfast and Clopidogrel™ at bedtime) to reduce possible interactions. *H. pylori* test and treat strategy may be beneficial for preventing gastroduodenal bleeding and its dangerous consequences in patients on double antiplatelet therapy after implantation of a coronary artery stent.
